# Effect of Pregnant Mothers' Forum Participation on Birth Preparedness and Complication Readiness among Pregnant Women in Dale District, Southern Ethiopia: A Comparative Cross-Sectional Study

**DOI:** 10.1155/2019/1429038

**Published:** 2019-05-05

**Authors:** Bizuayehu Bogale, Ayalew Astatkie, Negash Wakgari

**Affiliations:** ^1^Kangaroo Mother Care Project, College of Medicine and Health Sciences, Hawassa University, Hawassa, Ethiopia; ^2^School of Public Health, College of Medicine and Health Sciences, Hawassa University, Hawassa, Ethiopia; ^3^Department of Midwifery, College of Medicine and Health Sciences, Hawassa University, Hawassa, Ethiopia

## Abstract

**Background:**

Pregnant mothers' forum is the innovative intervention strategy being implemented in Ethiopia to facilitate birth preparedness and complication readiness practice. However, its effect on birth preparedness and complication readiness has not been investigated.

**Objective:**

This study assessed the association of participation in pregnant mothers' forum with birth preparedness and complication readiness plan among pregnant women in Dale District.

**Methods:**

A community-based comparative cross-sectional study was conducted among 604 pregnant women (302 who were forum members [exposed] and 302 who were forum nonmembers [unexposed]). Multistage sampling technique was used to select respondents. Data were collected door to door using a pretested and structured questionnaire through face-to-face interview. Data were entered and analyzed using SPSS version-20. Multiple logistic regression analysis was used to identify the effect of pregnant mothers' forum membership on birth preparedness and complication readiness adjusting for other variables.

**Results:**

About 22.5% of pregnant women were well prepared for birth. A quarter (25.8%) of the women was prepared for the anticipated complications of whom 20.7% were the forum members. Being pregnant mother's forum member (AOR=2.86, 95% CI=1.50,5.44), having focused counseling (AOR=3.73, 95% CI=1.17,11.83), monthly income (AOR=2.55, 95% CI=1.44,4.51), having antenatal care (AOR=3.73,95% CI=1.05,13.21), and institutional delivery during last birth (AOR=2.41, 95% CI=1.38,4.22) were significantly associated with birth preparedness. Similarly, being forum members (AOR=3.55, 95%CI=2.18, 5.78) and having antenatal care attendance before or at four months of gestational age (AOR=3.16, 95%CI=2.04, 4.91) were found to be predictors of complication readiness.

**Conclusion:**

In this study, birth preparedness and complication readiness is found to be low. However, it was significantly higher among forum members compared to forum nonmembers. Hence, efforts should be targeted to strengthen the pregnant mothers' forum and enroll the pregnant women to antenatal care service at early stage of the pregnancy.

## 1. Introduction

Worldwide, maternal mortality remains a public health challenge particularly in the developing regions [1, 2]. Global maternal mortality ratio decreased from 385 to 216 maternal deaths per 100,000 live births between 1990 and 2015 as a result of commitment of countries to reduce maternal deaths by 75%. However, the annual rate of decline was 2.3% between 1990 and 2015 which is far from 5.5% yearly target reduction [1]. Ethiopia is one among ten countries contributing for 59% of global maternal deaths [1, 2].

Birth preparedness and complication readiness (BPACR) is the process of planning for normal delivery and anticipating the actions needed in case of an emergency [3]. It helps to ensure that women can reach skilled delivery care when labor begins and reduce delay to seek care and to reach health care facilities that occur when women experience obstetric complications [3, 4]. In addition, it emanates from the fact that most pregnancy complications are sudden and unpredictable [5]. It promotes active preparation and decision making for delivery by pregnant women and their family [3–5]. In contrast, unprepared families will waste a great deal of time in recognizing problem, getting organized, getting money, finding transport, and reaching the appropriate health facility when complications occur [6, 7].

Pregnant mothers' forum has been carried out in Ethiopia as a strategy to facilitate preparedness for birth at health facility and seeking care early during an emergency. It is expected that every pregnant women regardless of gestational age should be a member of pregnant mothers' forum. The forum activity begins with identification of pregnant women jointly by community organizations (one-to-five networks and health development army) and health extension workers. Women enrolled to the forum are expected to attend continuously every two weeks whereby they take an opportunity to contact midwives or other high-level professionals in their kebeles (villages) concerning their pregnancy. The pregnant women also share their experiences to each other which further encourage them to decide to seek skilled health care. The role of skilled professionals preferably midwife is simply facilitation and guidance during the discussion and finally summarizing the issues raised for that particular session. The major topics to be covered during pregnant mothers' forum meetings include danger signs of obstetric complications, choosing preferred place and attendant at birth, making advance arrangement for transportation to reach skilled care site, saving money for incurred costs of skilled and emergency care, and finding who will accompany them at birth or in case of emergency care. Other measures include identifying compatible blood donor in case of emergency, obtaining consent from head of household to seek skilled care in the event the birth emergency occurs in his absence and arranges a source of support to provide a temporary family care during her absence [8].

A research finding showed that increasing awareness of pregnant women on birth preparedness and complications would rectify early detection of the obstetric complication and use of skilled care and reduces the delay [9–11]. However, the association of pregnant mothers' forum membership with BPACR has not yet been studied. Moreover, the overall status of birth preparedness and complications among pregnant women in the study area is also not well known. Hence, this study assessed the association of participation in pregnant mothers' forum with birth preparedness and complication readiness among pregnant women in Dale Woreda, Southern Ethiopia.

## 2. Materials and Methods

### 2.1. Study Setting and Population

The study was conducted in Dale Woreda, Sidama Zone, which is found in the Southern Nations, Nationalities, and Peoples Region (SNNPR) of Ethiopia. It is located at 328 km south west to the capital city of Ethiopia, Addis Ababa. The Woreda is divided into 36 rural administrative kebeles (villages). At the time of the study, the total population in the Woreda was estimated to be 272,203. The size of women of reproductive age group was estimated to be 23.3% of the total population (i.e., about 63,423). The estimated size of pregnant women in the Woreda was about 9,418 (3.46%) [Personal communication, Dale Woreda Health Office, 2015].

Regarding health facilities in the Woreda, it has one general hospital, ten health centers, thirty-six health posts, and three clinics (two owned by faith-based organizations (NGOs) and one private). The number of health professionals working in these public health institutions in the Woreda during the study was as follows: 14 first degree graduate nurses and health officers, 106 diploma clinical nurses, 11 midwives, 12 public health nurses, 25 medical laboratory technicians, 5 environmental health officers, and 16 pharmacists. In addition there were 81 health extension workers (HEWs) providing primary health care services in each kebele's health posts [Personal communication, Dale Woreda Health Office, 2015].

### 2.2. Study Design

A community-based comparative cross-sectional study design was conducted from December 1-28, 2015

### 2.3. Source and Study Population

All pregnant women in the Woreda were taken as source population. Pregnant women of three or more months of gestational age were enrolled as the study population. This is because mostly pregnancy is well understood and considered in the community around three months or later. Women, who were permanent residents of the area and voluntary to participate in the study, were included in the study. Pregnant women who were severely ill during the study period were excluded.

### 2.4. Sample Size Determination

Sample size calculation was made based on the assumptions that the total population estimated in the Woreda is 272, 203 and the proportion of expected pregnant women in the Woreda is 3.46% of the total population. With these assumptions, the total pregnant women in the Woreda were estimated to be 9,418. Accordingly, Epi-info version 7 software was used to calculate the required sample size with the two population proportion formula. Proportion of women prepared for child birth and enrolled to the pregnant women forum was assumed to be 50% due to the absence of a previous study based on which the proportion can be assumed. The proportion of birth preparedness among women not enrolled in the forum was taken to be 35% (i.e., a proportion difference of 15%; this is just an assumption as there is no previous study based on which this difference can be estimated). A 95% confidence level, an 80% power, and a ratio of unexposed to expose of 1 were considered. A nonresponse rate of 10% and design effect of 1.5 were also assumed. Based on the above assumptions, the required sample size becomes 604 (302 exposed [forum members] and 302 unexposed [nonforum members]).

### 2.5. Sampling Procedures

A multistage sampling technique was employed to enroll the study units. In the primary sampling stage, 11 kebeles were randomly selected (considering at least 30% of kebeles in the Woreda) using computer generated random numbers. The selected kebeles were Gajamo, Soyama, Moto, Gane, D/kege, Dagiya, Shafina, Wara, Shoye, Tula, and Semen Mesenkela. At the secondary stage, households with eligible women for the study were identified using preliminary survey. Preliminary survey was carried out by health promoters in each selected kebele one week before the actual data collection. The preliminary survey data collection tool contained the name of pregnant women, village (‘got'), whether or not attending the forum and attendance within a month, and phone number information. List of pregnant women who were enrolled and not enrolled in the pregnant mothers' forum were drawn from the lists obtained from preliminary survey and cross-checking with the record of health posts. Then, sample was proportionally allocated to each kebele considering the number of both forum and nonforum members. Then, individual women from the two sampling frames were drawn independently using systematic random sampling techniques with k^th^ value of two. For households which have more than one eligible woman, one woman was randomly selected using the lottery method. In the situation of absence of women from home during the survey, data collectors returned in the next two consecutive days and all respondents were interviewed.

### 2.6. Data Collection Tools and Procedures

A structured questionnaire adapted from Monitoring Birth Preparedness and Complication Readiness tools and Indicators for Maternal and Newborn Health manual formulated by Johns Hopkins Program for International Education in Gynecology and Obstetrics (JHPIEGO) in 2004 was used with modification to fit the context of the study area [12]. The variables addressed by the questionnaire include sociodemographic characteristics, obstetric history, and whether the women have taken basic BPACR actions: identified trained birth attendant, identified health facility for skilled delivery and emergency, identified mode of transport for skilled delivery and/or obstetric emergency, saved money, identified blood donor, and visited a higher health institution at least once for antenatal care during pregnancy. Knowledge of obstetric danger signs, intention of service use, awareness of newborn care, and danger signs questions related to participation in pregnant mothers' forum were also included in the tool. Questions in relation to participation in pregnant mothers' forum were also included in the questionnaire. The questionnaire was prepared in English and then translated to the local language (Sidaamu Afoo). Another individual who had good understanding of both Sidaamu Afoo and English translated the Sidaamu Afoo version back to English to check for its original meaning.

The questionnaire was pretested in a kebele that has similar characteristics with the selected kebeles. A total of 30 respondents (5% of sample size) were interviewed for the pretest. Findings of the pretest were discussed in the presence of data collectors and supervisors in order to ensure better understanding of the data collection process. Based on the pretest findings, questions were revised and edited, and those found to be unclear or confusing were modified. Finally, the structured close-ended Sidaamu Afoo version questionnaire was used for data collection. Eight diploma nurses who were fluent in Sidaamu Afoo language collected the data. Two supervisors with a first degree in midwifery supervised the data collectors. Two days training was given on the study instrument and data collection procedures. Data was collected through face-to-face interview with the study participants at their home. Filled questionnaires were checked daily for completeness, legibility, and consistency. Incomplete and unclear questionnaires were returned to the interviewers to get it completed by the next day.

### 2.7. Variable Definitions

A pregnant woman was considered to be prepared for birth if she could make arrangements on four of the following six key birth preparedness practices: identified place of delivery, identified skilled provider at child birth, saved money, identified a means of transportation, visited higher health facility at least once for ANC, and identified blood donor [4]. A woman was considered to be knowledgeable on danger signs during pregnancy if she could mention at least two of the three key danger signs for pregnancy (vaginal bleeding, swollen hands/face, and blurred vision) [13]. Similarly, a woman was declared to be knowledgeable for danger signs of labor/child birth if she could mention at least two of four key danger signs for labor/childbirth (sever vaginal bleeding, prolonged labor>12 hours, convulsion, and retained placenta) [13]. Moreover, a women was regarded as knowledgeable for obstetric danger signs of the postpartum period if she could mention at least two of three key danger signs of the postpartum period (sever vaginal bleeding, foul-smelling vaginal discharge, and high fever) [14]. A woman was considered to have complications readiness if she could spontaneously mention a total of three key danger signs in all three phases (during pregnancy, child birth, and postpartum periods) with at least one at each phase. A woman was considered to be aware of danger signs of new born if she can mention promptly two of four key danger signs of newborn (difficult/fast breathing, unable to feed, convulsion, and lethargy/unconsciousness). Besides, a woman was considered to be aware of new born cares if she could agree on two of three the following cares: early initiation of breast feeding, importance of colostrums, and exclusive breast feeding.

### 2.8. Data Management and Analysis Procedure

Data were entered, cleaned, and analyzed by the statistical packages for social sciences (SPSS version 20). Univariate analyses were conducted to obtain descriptive summaries. Data are presented in the form of texts, tables and graphs. Bivariate logistic regression was used to identify candidate variables for multiple logistic regression analysis. Multicollinearity between the independent variables was assessed by using variance inflation factors (VIF > 10 was considered as suggestive of multicollinearity) before interpreting the final output. However, no significant multicollinearity was detected as VIF for all variables were <5. Finally, multiple logistic regression analysis was used to determine the association between participation in pregnant mothers' forum and birth preparedness and complication readiness adjusting for confounding variables. Those variables with p-values less than or equal to 0.25 on bivariate analysis were included in the multiple logistic regression model. P-value and 95% confidence interval (CI) for the adjusted odds ratio (AOR) were used in judging the significance of the associations. P-values less than or equal to 0.05 were used to declare statistically significant associations.

## 3. Results

### 3.1. Sociodemographic Characteristics of the Study Participants

Out of the total 604 (302 forum members and 302 nonmembers) pregnant women planned for the study, all were enrolled in the study making a response rate of 100%. The mean (± standard deviation) of respondents' age was 26.5(±5.6), with range of 12-46 years. The mean age of forum member women was 26.56 years (95% confidence interval [CI]: 25.95, 27.19) and that of the forum nonmember was 26.52 years (95%CI: [25.92, 27.23]). Details of the sociodemographic characteristics of the study participants are given in Table 1.

### 3.2. Obstetric History and Antenatal Care Experience of the Respondents in Dale Woreda

Two hundred and fifty-eight (42.7%) of the respondents had already given birth to two to four children. Among the forum members, 53(8.8%) had delivered one child and 18(3.0%) had five or more children [x^2^=1.1, P< 0.77]. The highest parity was 7 among the forum members and 9 among forum nonmembers. Three hundred and eighty-two (63.2%) of the respondents were in the 3^rd^, 215 (35.6%) in the 2^nd^, and 7 (1.2%) in the 1^st^ trimester of pregnancy. Forty (6.6%) respondents had experienced history of stillbirth during last birth. Of these, 22 (3.6%) were among the forum members while the remainder 3.0% were among the forum nonmembers. Four hundred and forty-six (73.8%) of respondents had attended antenatal care during current pregnancy of whom 284 (47.0%) were forum members and 162 (26.8%) were forum nonmembers [x^2^=127.57, P< 0.001]. Among those pregnant women who attended ANC, nearly half 296 (49%) had visited once. Regarding timing for ANC, only 168 (27.8%) respondents visited health facility for first ANC before/at four months of gestational age. Among those women who started to visit health facility early for antenatal care, 119 (19.7%) were forum members [x^2^=40.4, P< 0.001] (Table 2). About 398 (65.9%) pregnant women planned to give birth at health facilities while 34.1% intended to deliver at their own home. Among those who planned for skilled deliver, 265 (43.9%) were forum members and 133 (22.0%) were forum nonmembers.

### 3.3. Birth Preparedness Practices

About three-fourth 456(75.5%) of the study participants had ever heard about birth preparedness. The women were asked about whether or not they were prepared for basic components of birth preparedness practices. Accordingly, 256 (42.4%) of pregnant women reported that they have arranged transportation to reach nearby health facility for skilled delivery or in case of emergency. More than half 327 (54.1%) of respondents identified health facility for skilled delivery. Of these, more than one-third 215 (35.6%) were forum members [x^2^=70.74, P< 0.001]. Considerable proportions 440 (72.8%) of the families have saved money for cost of delivery and emergency when needed. Only few 37 (6.1%) identified potential blood donor in case of emergency. Similarly, only 170 (28.1%) of pregnant women had visited higher health facilities (hospital/health center/clinic) at least once for antenatal care. Overall, one hundred and twenty-six (22.5%) of the pregnant women in this study were well prepared for birth. More than three quarter 468 (77.5%) were considered as less prepared. Among those women who were well prepared, 109 (18.0%) were forum members and 27 (4.5%) were forum nonmembers [x^2^=63.8, P< 0.001]. Only 22 (3.6%) of the study participants were well prepared for all six measures. Of these, 20 (3.3%) were forum members and 2 (0.3%) were forum nonmembers (Table 3).

### 3.4. Knowledge about Obstetric Danger Signs among Pregnant Women

One hundred and eleven (42.9%) of pregnant women were able to mention the obstetric danger signs: vaginal bleeding was the most; 217 (35.9%) mentioned obstetric danger sign during pregnancy. Similarly, severe headache 104 (17.2%), swelling of hands/face 82 (13.6%), severe weakness 52 (8.6%), high fever 51 (8.4%), blurring of vision 42 (7.0%), loss of consciousness 40 (6.6%), and convulsion 33 (5.5%) were the also mentioned danger signs by pregnant women. Vaginal bleeding 60 (9.9%), severe headache 27 (4.5%), swelling of hands/face 11 (1.8%), severe weakness 9 (1.5%), high fever 4 (0.7%), blurring of vision 5 (0.8%), loss of consciousness 6 (1.0%) and convulsion 5 (0.8%) were the mentioned danger signs by pregnant women from forum nonmembers. Moreover, vaginal bleeding 157 (26%), severe headache 77 (12.7%), swelling of hands/face 71 (11.8%), severe weakness 43 (7.1%), high fever 47 (7.8%), blurring of vision 37 (6.1%), loss of consciousness 34 (5.6%), and convulsion 28 (4.6%) were the mentioned danger signs by pregnant women from forum members.

### 3.5. Knowledge of Obstetric Danger Signs during Labor among Pregnant Women

Only 120 (44.5%) of the respondents were able to mention the obstetric danger signs of labor. Severe vaginal bleeding was the most 230 (38.1%) mentioned obstetric danger signs followed by severe headache 100 (16.6%). About 170 (28.1%) of the respondents among forum members mentioned severe vaginal bleeding as obstetric danger sign while only 60(10%) mentioned the danger sign among forum nonmembers. The key obstetric danger signs of childbirth mentioned were severe vaginal bleeding 230 (38.1%), retained placenta 68 (11.3%), prolonged labor 66 (10.9%), and convulsion 30 (5.0%) with decreasing order of the frequency. Only 170 (28.1%), 49 (8.1%), 46 (7.6%), and 23 (3.8%) of the forum members spontaneously mentioned the key danger signs as severe vaginal bleeding, prolonged labor, retained placenta, and convulsion, respectively. Similarly, 60 (10%), 22 (3.6%), 17 (2.8%), and 7 (1.2%) proportions among forum nonmembers mentioned severe vaginal bleeding, retained placenta, prolonged labor, and convulsion in the order given.

### 3.6. Knowledge of Obstetric Danger Signs of Postpartum Period among Pregnant Women

Overall 243 (40.2%) of respondents were able to explain the anticipated danger signs that occur immediately after delivery. Of these, 174 (28.8%) of the study participants were forum members. Severe vaginal bleeding was the most mentioned obstetric danger sign of postpartum 197 (32.6%) followed by severe headache 85 (14.1%). In addition, high fever 45 (7.5%), blurring of vision 25 (4.1%), severe abdominal pain 17 (2.8%), foul-smelling vaginal discharge 56 (9.3%), and loss of consciousness 21 (3.5%) were mentioned as key danger signs. About 145 (24%) of the respondents among the forum members mentioned severe vaginal bleeding as obstetric danger sign while only 52(8.6%) of pregnant women from nonforum members were able to mention severe vaginal bleeding as danger sign after delivery. Furthermore, high fever 42 (7.0%), blurring of vision 18 (3.0%), severe abdominal pain 11 (1.8%), foul-smelling vaginal discharge 44 (7.3%), and loss of consciousness 19 (3.1%) were mentioned as key danger signs after delivery by pregnant women from forum members, while high fever 3 (0.5%), blurring of vision 7 (1.2%), severe abdominal pain 6 (1.0%), foul-smelling vaginal discharge 12 (2.0%), and loss of consciousness 2 (0.3%) were mentioned as key danger signs after delivery by pregnant women from nonforum members.

### 3.7. Overall Complication Readiness among Pregnant Women

Overall, 156 (25.8%) of the respondents were well aware of the anticipated complications in all three phases for the current pregnancy and thus were categorized having complication readiness. Of these, most 125 (20.7%) of the complication readiness was among the forum members [x^2^=76.3, P< 0.001] (Table 4).

### 3.8. Knowledge about Danger Signs of Newborn and Newborn Care Practices

Only 84 (13.9%) of the pregnant women were considered as knowledgeable for newborn danger signs. Of these, 72 (11.9%) were forum members. Difficulty/fast breathing 105 (17.4%), jaundice (yellow skin/eye) 22 (3.6%), poor sucking/feeding 125 (20.7%), convulsion 18 (3.0%), unconsciousness 24 (4.0%), bleeding/pus discharge from umbilical cord 29 (4.8%), and skin lesion (pustules) 7 (1.2%) were newborn's danger signs mentioned by pregnant women (Table 5). Moreover, a considerable proportion, 535 (88.6%), of the respondents was aware of early initiation of breast feeding. Among those women 283 (46.9%) were forum members. Three-quarters, 453 (75.0%), of respondents had knowledge of the importance of giving colostrums for newborn baby. Of these, 251 (41.7%) were forum members. Furthermore, 524 (86.8%) of pregnant women had knowledge for exclusive breast feeding. Among these, 282 (46.7%) were forum members. Overall 521 (86.3%) of the respondents were aware of at least two of the three important breast feeding practices. Of these, 284 (47.0%) were forum members.

### 3.9. Factors Influencing Practice of Birth Preparedness in Dale Woreda

On crude logistic regression, pregnant mother's forum membership, educational level of pregnant women, average monthly income, family size, birth place of during the last delivery, counseling about BPACR, ANC follow-up, knowledge of danger signs during pregnancy, labor, and postpartum were independently tested and found to have statistically significant association with birth preparedness. After controlling for possible confounders, the adjusted multivariable model showed being members of pregnant mother's forum (AOR=2.86, 95% CI=1.50,5.44), having received counseling women focused on birth preparedness (AOR=3.73, 95% CI=1.17,11.83), monthly income >301 birr (AOR=2.55, 95% CI=1.44,4.51), ANC follow-up (AOR=3.73, 95% CI=1.05,13.21), institutional delivery during last birth (AOR=2.41, 95% CI=1.38,4.22), having knowledge of at least two danger signs during pregnancy (AOR=2.24, 95% CI=1.08,4.64), and having knowledge of at least two danger signs of childbirth (AOR=2.21, 95% CI=1.16,4.20) to have statistically significant association with birth preparedness. The odds of birth preparedness among forum members were nearly three times higher compared to forum nonmembers (AOR=2.86, 95% CI=1.50, 5.44).

The odds of birth preparedness for current pregnancy among those women who had ANC follow-up were nearly four times higher when compared to those women who did not have ANC follow-up (AOR=3.7, 95% CI=1.03, 13.1). Furthermore, birth preparedness among the pregnant women who gave birth at health facility during last birth was significantly higher compared to those who had delivered at home (AOR=2.4, 95% CI=1.38, 4.19) (Table 6).

### 3.10. Factors Influencing the Complication Readiness in Dale Woreda

On crude logistic regression analysis forum membership, educational level of pregnant women, family size, ANC attendance, timing for first ANC, focused counseling on birth preparedness and complication readiness, and place for first ANC were independently tested and found to have statistically significant association with complication readiness. However, on multivariable logistic regression analysis controlling for possible confounders, forum membership status (AOR=3.55, 95% CI=2.18, 5.78), ANC visit before or at four months of gestational age (AOR=3.16, 95% CI=2.04, 4.91), and having received focused counseling on BPACR (AOR=3.66, 95% CI=1.65, 8.10) were statistically significantly associated with complication readiness.

The odds of complication readiness among forum members were more than three times higher compared to those women who were forum nonmembers (AOR=3.55, 95% CI=2.18, 5.78). In addition, those pregnant women who started first antenatal care before/at gestational age four months were found to have three times higher odds of readiness for the anticipated complications (AOR=3.06, 95% CI=1.86,5.01). Furthermore, the odds of complication readiness among those pregnant women who started attendance for antenatal services in health posts were 3.24 times higher compared to those who visited health center/hospital for ANC (AOR=3.24, 95% CI=1.24, 8.46) (Table 7).

## 4. Discussion

In this study, the prevalence of birth preparedness was found to be 22.5% [CI: 20.4-24.7] and that of complication readiness to be 25.8% [CI: 23.5-26.3]. The finding of this study is in agreement with the finding of the study conducted in Adigrat town, North Ethiopia (22%) [15]. This finding is higher than the finding of a study in Duguna Fango, South Ethiopia (18.3%) [16]; however, it is lower compared with the studies done in Goba, East Ethiopia (29.9%) [7], and Indore city, India, 47.8% [17]. This could be due to the difference in the local context (sociocultural characteristics) and the difference in implementation of maternal health programs. Besides, it could be due to differences in the number of birth preparedness components considered in those studies to declare as prepared for the birth. For instance, four of six components of birth preparedness in the current study, four of five in Goba Woreda, and two of three in Indore city, India, were used to declare a woman as well prepared for birth. In addition, the present study revealed that 18.0% of forum members and 4.5% of forum nonmembers were well prepared for birth. Also 20.7% of forum members and 5.1% of forum nonmembers were well aware of the obstetric complications and thus expected to seek timely health care during emergency. The difference in proportions between the two groups clearly indicates that being forum members increases the likelihood of BPACR among pregnant mothers.

Further, being member of pregnancy mother's forum was found to be a strong predictor for both birth preparedness and complication readiness. Those pregnant women enrolled to the forum were nearly three to four times more likely to be prepared for birth and complications than those women who were not ever enrolled. The reason for this might be due to the fact that participation in the pregnancy mothers' forum gives more opportunity for pregnant women to be informed and educated about BPACR in detail. Health professionals may have established trust and positive attitude among the pregnant women enrolled to the forum for health facility delivery than women not enrolled to the forum. Unlike the usual trend of individual counseling, educating mothers in mass as in the pregnant mothers' forum creates chance of sharing their past experiences from each other. This is important for the women to learn more practically other than simply to tell them to do so and thus to have increased risk perception which is vital to plan for skilled health care. Besides, exploring the individual barriers of the utilization of the skilled health care is important to make appropriate plan in the short or long run to mitigate the problems on the spot. On the other hand, this implies that participation in pregnant mothers' forum is important to maximize the responsibility of the community via participation in planning and acting together in the efforts of improving maternal health.

Complications readiness among the forum members is relatively higher compared to birth preparedness. The possible explanation for this may be that those in an earlier stage of pregnancy might not yet have made arrangement for delivery. Such women would still have opportunity for birth arrangements in line with their good understanding of the obstetric complications. The comprehensive knowledge of obstetric danger signs among pregnant women in the study area was found to be low, but relatively higher than that of the prevalence of birth preparedness which is inconsistent with study done in southeast Nigeria [18].

This study showed ANC follow-up to be a predictor of birth preparedness and complication readiness. This evidence is consistent with studies conducted in Aleta wondo, Duguna Fango, and India [3, 16, 17]. Another important finding of this study was the fact that timing of first ANC visit was found to be an important predictor of complication readiness. Those women who started ANC before or at gestational age of 4 months were three times more likely to be ready for complications than those who started ANC follow-up after 5 months of gestational age. This may be because they would have enough duration for arrangements and would have increased chance to meet health workers and have discussion concerning the current pregnancy. Thus, pregnant mother's forum may also have played important role to identify pregnancy as early as possible and to link them to the necessary health services.

The other key finding of this study is that those pregnancy women who attended first ANC in health posts are more likely to be knowledgeable for the anticipated complications than those who attended in health centers or hospitals. This is might be due to health extension workers in health posts taking enough time and giving due attention to educate mothers on obstetric complications with their own culturally tailored approaches than those health professionals working in health centers or hospital. This indicates that, instead of providing the clinical service only, taking time and giving attention to counsel pregnant women on BPACR are important to increase BPACR practice.

The evidence in this research showed that women with monthly income of above the median value (>300 birr) were found to have higher birth preparedness relative to those with monthly income of less than the median value. The finding is not consistent with the research conducted in Robe Woreda, central Ethiopia, which reported those with a monthly income of >716 birr to have higher birth preparedness [4]. The possible reason for this might be the difference in agroecology. The seasonal cash crop (coffee) is the predominant source of family income in the study area while cereals and vegetables are continuous source of family income in Robe. This implies that efforts to increase BPACR may also need to focus on strategies that enable pregnant women to collectively produce their own income and use it for delivery or emergency services in addition to the current effort made to make maternal health services free of charge. This calls the need for collaborative effort of health sector to work with local small scale microfinance institutions. Working with the community structural organizations (one-to five networks and health developing army) to mobilize the resources is also important.

This study also showed that institutional delivery during last birth has influenced BPACR. Those women who had given birth in the health facility during last birth were more likely to be prepared for birth of the current pregnancy than those who delivered at home. The finding is in agreement with the study conducted in Goba District [9]. This could be because those who had delivered in the facility have better health seeking behavior than those who had delivered at home during last birth and thus may also be well prepared for birth of the current pregnancy. Similarly, women who had knowledge of at least two key danger signs during pregnancy were more likely to be well prepared, which is consistent with studies done in Duguna Fango [16] and Uganda [19]. Besides, women who had knowledge of at least two key danger signs of childbirth were more likely to be prepared for birth. The finding is consistent with study conducted in Tanzania [20]. This implies that the knowledge of obstetric complications is essential for women to prepare for birth.

Moreover, the knowledge of the women on newborn complications in the study area was found to be 13.9%. This indicates lack of due attention on educating the women on newborn danger signs. However, those pregnant women who were forum members were found to have relatively better awareness of newborn danger signs and newborn cares than those women who were forum nonmembers. This shows that the interventions targeted to improve BPACR should consider the equal importance of enhancing awareness of the women for the newborn complications as part of birth preparedness and complication readiness.

This study has some limitations. Firstly, recall bias cannot be excluded. Those pregnant women in both groups who failed to mention any of the key obstetric danger signs promptly may be due to the recall problem. Secondly, since the study participants have not completed their pregnancy, they may not yet have had the opportunity or need to make arrangements related to BPACR. This may result in an underestimation of the prevalence of birth preparedness practices.

In conclusion, this study showed BPACR among pregnant women in Dale Woreda to be low. However, BPACR was significantly higher among pregnant women who were forum members compared to forum nonmembers. Hence, efforts should be targeted to strengthening the pregnant mothers' forum and enrolling the pregnant women to antenatal care service at early stage of the pregnancy.

## Figures and Tables

**Table 1:** Sociodemographic characteristics of the study participants in Dale Woreda, Sidama Zone, South Ethiopia, 2015 (n=604).

Variables	Total (%)	Forum members	Forum non-members	Pearson chi-square	p-value
		Number (%)	Number (%)		
Age in years					
< 20	93(15.4)	46(7.6)	47(7.8)		
21-25	202(33.4)	103(17.1)	99(16.4)	1.94	0.58
26-30	138(22.8)	73(12.1)	65(10.8)		
31-34	106(17.5)	47(7.8)	59(9.8)		
>35	65(10.8)	33(5.5)	32(5.3)		
Marital status					
Married	599(99.2)	299(49.5)	300(49.7)	Fishers' exact test	1.0
Others*∗*	5(0.8)	3(0.5)	2(0.3)		
Religion					
Protestant	519(85.9)	257(42.5)	262(43.4)		
Muslim	49(8.1)	27(4.5)	22(3.6)	0.68	0.87
Orthodox	23(3.8)	11(1.8)	12(2.0)		
Catholic	13(2.2)	7(1.2)	6(1.0)		
Ethnicity					
Sidama	579(95.9)	285(47.2)	294(48.70)	Fishers' exact test = 3.47	0.17
Amhara	17(2.8)	12(1.98)	5(0.82)		
Wolayita	8(1.3)	5(0.8)	3(0.5)		
Educational level					
No	91(15.1)	41(6.8)	50(8.3)		
First cycle (1-4)	186(30.8)	81(13.4)	105(17.4)	9.93	0.019
Second cycle (5-8)	280(46.4)	149(24.7)	131(21.7)		
9^th^ and above	47(7.8)	31(5.1)	16(2.7)		
Occupation					
Housewife	496(82.1)	234(38.7)	262(43.4)	Fishers' exact test = 8.94	
Merchant	103(17.1)	65(10.8)	38(6.3)		0.007
Others*∗∗*	5(0.8)	3(0.5)	2(0.3)		
Family income					
<500	495(82.0)	237(39.2)	258(42.7)	Fishers' exact test = 5.04	0.078
501-1000	103(17.0)	61(10.1)	42(7.0)		
>1000	6(1.0)	4(0.7)	2(0.3)		
Partner occupation					
Farmer	338(56.0)	148(24.5)	190(31.5)		
Private employee	30(5.0)	15(2.5)	15(2.5)	17.63	0.001
Gov. employee	15(2.5)	13(2.2)	2(0.3)		
Merchant	221(36.6)	126(20.9)	95(15.7)		
Partner's education					
No	42(6.9)	20(3.3)	22(3.6)		
Read and write	57(9.4)	32(5.3)	25(4.1)	2.01	0.73
1^st^ cycle (1-4)	160(26.5)	78(12.9)	82(13.6)		
2^nd^ cycle (5-8)	243(40.2)	117(19.4)	126(20.8)		
9^th^ and above	102(16.9)	55(9.1)	47(7.8)		
Family size					
≤ 4	383(63.4)	208(34.4)	175(29.0)		
5-6	127(27.6)	73(12.1)	94(15.6)	8.15	0.017
≥ 7	50(8.9)	21(3.5)	33(5.5)		

*Note.*  *∗* single, widowed, and divorced, *∗∗*occupations like private and government employment.

**Table 2:** Obstetric history and antenatal care experience of the respondents in Dale Woreda, 2015.

Variables	Total (%)	Forum members	Forum non-members	chi-square	p-value
		Number (%)	Number (%)		
Gravid					
Primi (1^st^)	179(29.6)	89(14.7)	90(14.9)		
2-3	258(42.7)	123(20.4)	135(22.3)	1.57	0.45
≥ 4	167(27.6)	90(14.9)	77(12.7)		
Parity					
Para 0	180(29.8)	90(14.9)	90(14.9)		
Para 1	101(16.7)	53(8.8)	48(7.9)	1.10	0.77
Para(2-4)	281(46.5)	141(23.3)	140(23.2)		
Para ≥5	42(7.0)	18(3.0)	24(4.0)		
History of still birth					
Yes	40(6.6)	22(3.6)	18(3.0)	0.43	0.51
Gestational age					
3-6 months	222(36.8)	107(17.7)	115(19.1)		
7-9 months	382(63.2)	195(32.3)	187(30.9)	0.45	0.50
Trimester during interview					
First	7(1.2)	0	7(1.2)		
Second	215(35.6)	107(17.7)	108(17.9)	7.17	0.03
Third	382(63.2)	195(32.3)	187(30.9)		
Antenatal care follow-up					
Yes	446(73.8)	284(47.0)	162(26.8)		
No	158(26.2)	18(3.0)	140(23.2)	127.57	0.001
Number of ANC visits (n=447)					
One	296(49.0)	103(17.0)	193(32.0)		
Two to three	285(47.2)	182(30.2)	103(17.0)	54.5	< 0.001
Four and above	23(3.8)	17(2.8)	6(1.0)		
GA during first ANC visit					
≤ 4 months	168(27.8)	119(19.7)	49(8.1)	40.4	< 0.001
≥ 5 months	436(72.2)	183(30.3)	253(41.9)		
Place for first ANC visit					
Health posts	177(39.7)	116(19.2)	61(20.5)		
Health centers	243(54.5)	155(34.7)	88(18.8)	129.7	< 0.001
Clinics/hospital	26(5.8)	13(2.9)	13(2.9)		

**Table 3:** Birth preparedness practices among pregnant women in Dale Woreda, 2015(n=604).

Level of birth preparedness practices	Total (%)	Forum members Number (%)	Forum non-members Number (%)	Chi-square*∗*	p-value
Arranged transportation					
Yes	256(42.4)	167(27.6)	89(14.7)	41.25	< 0.001
No	348(57.6)	135(22.4)	213(35.3)		
Identified skilled birth attendant					
Yes	107(17.7)	91(15.1)	16(2.6)	63.88	< 0.001
No	497(82.3)	211(34.9)	286(47.4)		
Identified facility for skilled delivery					
Yes	327(54.1)	215(35.6)	112(18.5)	70.74	< 0.001
No	277(45.9)	87(14.4)	190(31.5)		
Saved money					
Yes	440(72.8)	249(41.2)	191(31.6)	28.15	< 0.001
No	164(27.2)	53(8.8)	111(18.4)		
Identified blood donor					
Yes	37(6.1)	33(5.5)	4(0.7)	24.21	< 0.001
No	567(93.9)	269(44.5)	298(49.3)		
Visited higher health facility for ANC at least once					
Yes	170(28.1)	110(18.2)	60(9.9)		
No	434(71.9)	192(31.8)	242(40.1)	20.5	< 0.001
Number of steps(arrangements)taken					
0	114(18.9)	34(5.6)	80(13.2)	22.8	< 0.001
1	123(20.4)	30(5.0)	93(15.4)	40.5	< 0.001
2	92(15.2)	49(8.1)	43(7.1)	0.46	0.49
3	139(23.0)	80(13.2)	59(9.8)	4.12	0.04
4	89(14.7)	68(11.3)	21(3.5)	29.1	< 0.001
5	25(4.1)	21(3.5)	4(0.7)	12.0	0.001
6	22(3.6)	20(3.3)	2(0.3)	15.2	< 0.001
Well prepared (>=4 arrangements made)	126(22.5)	109(18.0)	27(4.5)	63.8	< 0.001
Not well prepared (< 4 arrangements)	468(77.5)	193(32.0)	275(45.5)		

Multiple responses were possible, *∗*= Pearson chi-square values were used.

**Table 4:** Comprehensive knowledge of obstetric danger signs among pregnant women in Dale Woreda, Sidama Zone, SNNPR, Ethiopia, 2015.

Variables	Number (%)	Forum members	Forum non-members	Chi-square	p-value
		Number (%)	Number (%)		
Knowledge on key danger signs of pregnancy					
No any danger signs	345(57.1)	116(19.2)	229(37.9)	88.3	<0.001
At least one danger sign	235(38.9)	171(28.3)	64(10.6)	79.7	<0.001
At least two danger signs	89(14.7)	77(12.7)	12(2.0)	55.6	<0.001
Three key danger signs	17(2.8)	17(2.8)	0	17.4	<0.001
Knowledge on key danger signs of labor					
No any danger signs	335(55.5)	114(18.9)	221(36.6)	76.7	<0.001
At least one danger sign	257(42.5)	179(29.6)	78(12.9)	69.0	<0.001
At least two danger signs	111(18.4)	86(14.2)	25(4.2)	41.0	<0.001
Three key danger signs	23(3.8)	20(3.3)	3(0.5)	13.0	<0.001
Knowledge on key danger signs of postpartum					
No any danger signs	361(59.8)	128(21.2)	233(38.6)	75.9	<0.001
At least one danger sign	213(35.3)	157(26.0)	56(9.3)	73.9	<0.001
At least two danger signs	81(13.4)	69(11.4	12(2.0)	46.3	<0.001
Three key danger signs	9(1.5)	9(1.5)	0	9.1	0.003
Overall complication readiness	156(25.8)	125(20.7)	31(5.1)	76.3	<0.001

**Table 5:** Knowledge about danger signs of newborn among pregnant women in Dale Woreda, Sidama Zone, SNNPR, Ethiopia, 2015.

Variables	Total (%)	Forum members	Forum non-members	Chi-square	p-value
		Number (%)	Number (%)		
Difficulty/fast breathing	105(17.4)	84(13.9)	21(3.5)	45.7	<0.001
Jaundice(yellow skin/eye)	22(3.6)	17(2.8)	5(0.8)	6.8	0.009
Poor sucking/feeding	125(20.7)	101(16.7)	24(4.0)	59.8	<0.001
Convulsion	18(3.0)	16(2.6)	2(0.4)	11.2	0.001
Unconsciousness	24(4.0)	18(3.0)	6(1.0)	6.2	0.012
Bleeding/pus discharge from umbilical cord	29(4.8)	21(3.5)	8(1.3)	6.0	0.014
Skin lesion(pustules)	7(1.2)	5(0.8)	2(0.3)	1.3	0.254

**Table 6:** Factors influencing birth preparedness adjusted for confounding variables, in Dale District, 2015.

Characteristics	Birth preparedness		Crude OR	Adjusted OR
	Less prepared	Well prepared	95% CI	95% CI
*Forum membership*	Number (%)	Number (%)		
Member	193(32.0)	109(18.0)	5.75(3.63-9.11)	*2.86(1.50-5.44)*
Non-member	275(45.5)	27(4.5)	1.0	1.0
*Education level*				
No education	79(13.1)	12(2.0)	0.13(0.58-0.30)	0.52(0.15-1.84)
Primary(1-8)	367(60.8)	99(16.4)	0.23(0.12-0.43)	0.74(0.26-2.13)
9^th^ and above	22(3.6)	25(4.1)	1.0	1.0
*Monthly income*				
≤300 birr	275(45.5)	64(10.6)	1.0	1.0
>300birr	193(32.0)	72(11.9)	1.60(1.09-2.35)	*2.55(1.44-4.51)*
*Family size*				
≤ 4	327(54.1)	100(16.6)	1.60(0.73-3.53)	2.37(0.91-6.14)
5-6	99(16.4)	28(4.6)	1.48(0.62-3.52)	1.61(0.58-4.45)
>7	42(7.0)	8(1.3)	1.0	1.0
*Antenatal care follow-up*				
Yes	315(52.2)	131(21.7)	12.72(5.1031.73)	*3.73(1.05-13.21)*
No	153(25.3)	5(0.8)	1.0	1.0
*Place of delivery during last birth*				
Health facility	71(16.7)	48(11.3)	3.47(2.16-5.58)	*2.41(1.38-4.22)*
Home	257(60.3)	50(11.7)	1.0	10
*Focused counseling on birth preparedness *				
Yes	329(54.5)	127(21.0)	5.96(2.94-12.06)	*3.73(1.17-11.83)*
No	139(23.0)	9(1.5)	1.0	1.0
*Aware of at least two key danger signs of pregnancy*				
Yes	47(7.8)	42(7.0)	4.0(2.49-6.41)	*2.24(1.08-4.64)*
No	421(69.7)	94(15.6)	1.0	1.0
*Aware of at least two key danger signs of labor*				
Yes	61(10.1)	50(8.3)	3.87(2.49-6.02)	*2.21(1.16-4.20)*
No	407(67.4)	86(14.2)	1.0	1.0
*Aware of at least two key danger signs after delivery*				
Yes	46(7.6)	35(5.8)	3.17(1.94-5.19)	1.02(0.48-2.15)
No	422(69.9)	101(16.7)	1.0	1.0

**Table 7:** Selected characteristics influencing the complication readiness among pregnant women in Dale District, Sidama Zone, SNNPR, 2015 (n=604).

Variables	Number (%) Complication readiness		Crude OR	Adjusted OR
	Yes	No	95% CI	95% CI
*Forum membership *				
Members	125(20.7)	177(29.3)	6.17(3.99-9.55)	3.55(2.18-5.78)*∗∗*
Non-members	31(5.1)	271(44.9)	1.0	1.0
*Education level*				
No education	19(3.1)	72(11.9)	0.38(0.18-0.84)	0.60(0.24-1.45)
Primary(1-8)	118(19.5)	348(57.6)	0.50(0.26-0.92)	0.76(0.37-1.55)
9^th^ and above	19(3.1)	28(4.6)	1.0	1.0
*Family size*				
1-2	28(5.7)	105(21.4)	1.34 (0.83-2.16)	1.18(0.75-1.85)
≥ 4	94(19.2)	263(53.7)	1.0	1.0
*Antenatal care follow-up*				
Yes	150(24.8)	296(49.0)	12.83(5.54-29.71)	2.28(0.86-6.01)
No	6(1.0)	152(25.2)	1.0	1.0
*Place for first antenatal visit*				
No antenatal visit	6(1.0)	152(25.2)	1.0	1.0
Health posts	66(10.9)	111(18.4)	15.0(6.30-35.98)	3.24(1.24-8.46)
Health center/hospital	84(13.9)	185(30.6)	11.5(4.88-27.0)	2.77(1.08-7.08)
*Gestational age during first antenatal care*				
Before/at 4 months	88( 14.6)	80(13.2)	5.95(3.99-8.86)	3.16(2.04-4.91)*∗∗*
Five or more months	68(11.3)	368(60.9)	1.0	1.0
*Focused counseling on BPACR*				
Yes	148(24.5)	308(51.0)	8.40(4.01-17.60)	3.66(1.65-8.10)
No	8(1.3)	140(23.2)	1.0	1.0

*Note.*  *∗∗* refers to the significance level of p-values less than 0.001.

## Data Availability

All data and materials in this manuscript could be deposited in any publicly available repositories.
